# T cell exhaustion assessment algorism in tumor microenvironment predicted clinical outcomes and immunotherapy effects in glioma

**DOI:** 10.3389/fgene.2022.1087434

**Published:** 2022-12-02

**Authors:** Lie Chen, Biao Fu

**Affiliations:** ^1^ Department of Neurosurgery, The People’s Hospital of Pingyang, Wenzhou, China; ^2^ Department of Neurosurgery, The People Hospital of Xinchang, Xinchang, China

**Keywords:** glioma, T cell exhaustion, immunotherapy, prognosis prediction, tumor environment

## Abstract

Despite the recent increase in the use of immune checkpoint blockade (ICB), no ICB medications have been approved or are undergoing large-scale clinical trials for glioma. T cells, the main mediators of adaptive immunity, are important components of the tumor immune microenvironment. Depletion of T cells in tumors plays a key role in assessing the sensitivity of patients to immunotherapy. In this study, the bioinformatics approach was applied to construct T cell depletion-related risk assessment to investigate the impact of T cell depletion on prognosis and ICB response in glioma patients. The Cancer Genome Atlas (TCGA) and GSE108474 glioma cohorts and IMvigor210 immunotherapy datasets were collected, including complete mRNA expression profiles and clinical information. We used cell lines to verify the gene expression and the R 3.6.3 tool and GraphPad for bioinformatics analysis and mapping. T cell depletion in glioma patients displayed significant heterogeneity. The T cell depletion-related prognostic model was developed based on seven prognostic genes (*HSPB1, HOXD10, HOXA5, SEC61G, H19, ANXA2P2, HOXC10*) in glioma. The overall survival of patients with a high TEXScore was significantly lower than that of patients with a low TEXScore. In addition, high TEXScore scores were followed by intense immune responses and a more complex tumor immune microenvironment. The “hot tumors” were predominantly enriched in the high-risk group, which patients expressed high levels of suppressive immune checkpoints, such as *PD1, PD-L1*, and *TIM3*. However, patients with a low TEXScore had a more significant clinical response to immunotherapy. In addition, *HSPB1* expression was higher in the U251 cells than in the normal HEB cells. In conclusion, the TEXScore related to T cell exhaustion combined with other pathological profiles can effectively assess the clinical status of glioma patients. The TEXScore constructed in this study enables the effective assessment of the immunotherapy response of glioma patients and provides therapeutic possibilities.

## Introduction

Despite aggressive treatment, gliomas are among the least successful primary tumors of the central nervous system, with a 5-year survival rate of less than 5% (Miller et al., 2021). Loss of effector function and increase in immunosuppressive molecules are the main features of T cell exhaustion (Dominguez-Villar et al., 2019; Choe et al., 2021). Indeed, it is common for CD4^+^ and CD8^+^ T cell numbers to be reduced within the tumor and in circulation among patients with glioma (Wang et al., 2017; Levite, 2021). As checkpoint inhibitors are witnessing an expanded range of indications, an increasing number of patients are receiving them (Zeng et al., 2013; Giles et al., 2018). Although PD-1 antibodies have been found to produce durable clinical responses in advanced melanoma, bladder cancer, liver cancer, and other types of cancer, it is unclear whether this treatment is feasible in glioma, suggesting the necessity to further investigate the mechanisms of T cell depletion in glioma (Farhood et al., 2019; Hu et al., 2019; Zheng et al., 2020; Laumont et al., 2021; Ye et al., 2021).

Various subtypes of T cells have been shown to play different roles in gliomas, primarily by mediating adaptive immune responses (Flores-Toro et al., 2020; Choi et al., 2021). Among them, T-regs and Th2 cells suppress antitumor immunity, whereas CD8^+^ and CD4^+^ T cells act as cytotoxic antitumor immune cells. Indeed, the tumor-killing capacity of many T cells of this kind may be suppressed in gliomas; as the tumor microenvironment becomes more complex, the prognosis becomes more inferior. It has been shown to be closely related to the tumor microenvironment, immunotherapy, and tumor prognosis (Du Four et al., 2016; Lowther et al., 2016; Jahan et al., 2018). With a large body of studies currently investigating the mechanism of T cell depletion on tumor progression and the causes of T cell immunosuppression in tumors, the overall relationship between T cell depletion and prognosis in glioma, as well as between the tumor microenvironment and immunotherapy sensitivity, remains unclear.

In our investigation, risk assessment related to T cell depletion in glioma, an independent factor for clinical outcomes, was developed using large-scale bioinformatics analysis. Patients with glioma were also examined for the relationship between clinical progression and T cell depletion to uncover the underlying mechanisms driving tumor progression.

## Materials and methods

### Data collection

Gene transcriptome data for low-grade gliomas (LGGs) and glioblastomas (GBMs) were collected from The Cancer Genome Atlas (TCGA), GSE108474 was downloaded as background corrected and RMA-normalized data, and the IMvigor 210 cohort was used as an immunotherapy cohort to verify clinical treatment outcomes. Among them, TCGA-LGG included a total of 515 samples’ transcriptome and clinical data, and TCGA-GBM included a total of 599 samples’ transcriptome and clinical data.

All transcriptome profiles were converted to transcripts per million (TPM) prior to analysis. Transcriptome data obtained from different platforms were corrected using the normalizeBetweenArrays function. Finally, transcriptome matrix data were screened to calculate the average value of duplicated genes.

### Assessment of immune cell infiltration

CIBERSORTx is a suite of machine learning tools for assessing cell abundance and cell-type-specific gene expression patterns from a large set of tissue transcriptome profiles (Steen et al., 2020). QuanTIseq is a method to quantify the tumor immune background as determined by the type and density of tumor-infiltrating immune cells (Finotello et al., 2017). TIMER explores the association between immune infiltration and a wide range of factors by performing predictive calculations of the levels of tumor-infiltrating immune subsets of cancer types with a comprehensive study of the molecular characteristics of tumor-immune interactions (Li et al., 2017). CIBERSORTx, QuanTiseq, and TIMER were employed to analyze the cell infiltration in TCGA samples, and the immune cell infiltration from TCGA data was investigated to identify the relationship between infiltrated immune cells, especially CD4^+^ and CD8^+^ T cells, and overall survival (OS) of glioma patients, with *p* < 0.05 being the entry for further analysis.

### Gene set enrichment analysis

The Gene Set Enrichment Analysis (GSEA) algorithm was applied to perform biological pathway enrichment between the two groups with Hallmark, Gene Ontology (GO), and Kyoto Encyclopedia of Genes and Genomes (KEGG) reference gene sets.

### Weighted correlation network analysis

Weighted correlation network analysis (WGCNA) can be used to describe the correlation between candidate features and gene sets. WGCNA was conducted using the R package WGCNA, and to ensure scale-free topological networks, the power value was set to *β* = 3 and the soft threshold parameter of scale-free networks to R2 = 0.9. Seven modules were retrieved as a result, with the green module displaying the closest relationship to the clinic for further study.

### Construction of TEXScore model

Random forest analysis was first performed on the selected genes, with the initial nTree = 1,000. When the error rate reached the minimum at nTree = 859, seven genes with relative importance greater than 0.2 were extracted for analysis. LASSO regression analysis was applied to the model equation, as follows:

$$risk\;score={Coef}_{1}\times {Gene\;expression}_{1}＋{Coef}_{2}\times {Gene\;expression}_{2}＋\cdots {Coef}_{n}\times {Gene\;expression}_{n}$$

*Coef* represents the importance index for each gene in the analysis random forest analysis. Gene expression values represent the expression values of the corresponding genes. For the assigned risk score, patients above the median risk score were defined as the high-risk group, whereas those below the median risk score were defined as the low-risk group. ROC and KM curves were used to assess model performance.

### Cell culture

GBM cell lines (U251) and normal human astrocyte cell lines (HEB) were obtained from Dr. Cai and Dr. Chen, respectively. The cells were seeded in RPMI-1640/DMEM supplemented with 10% fetal bovine serum (Gibco, China) at 37°C in a 5% CO_2_ atmosphere.

### Quantitative real-time PCR

Cells were treated with TRIzol reagent (Takara, Japan). We then extracted all RNA and reverse-transcribed it into cDNA. qRT-PCR was used to analyze the relative expression of HSPB1, and data were normalized to GAPDH. The primers used are listed in Supplementary Table S1.

### Statistical analysis

All data analysis results in our investigation were processed using R software. For continuous variables with normal distribution, we conducted Student’s *t*-test, while non-normally distributed continuous variables were calculated using the Mann–Whitney U test. Differential expression analysis was conducted with a threshold set at |logFC| > 0.5 and *p* < 0.05.

## Results

### T cell-related model construction

**FIGURE 1:**
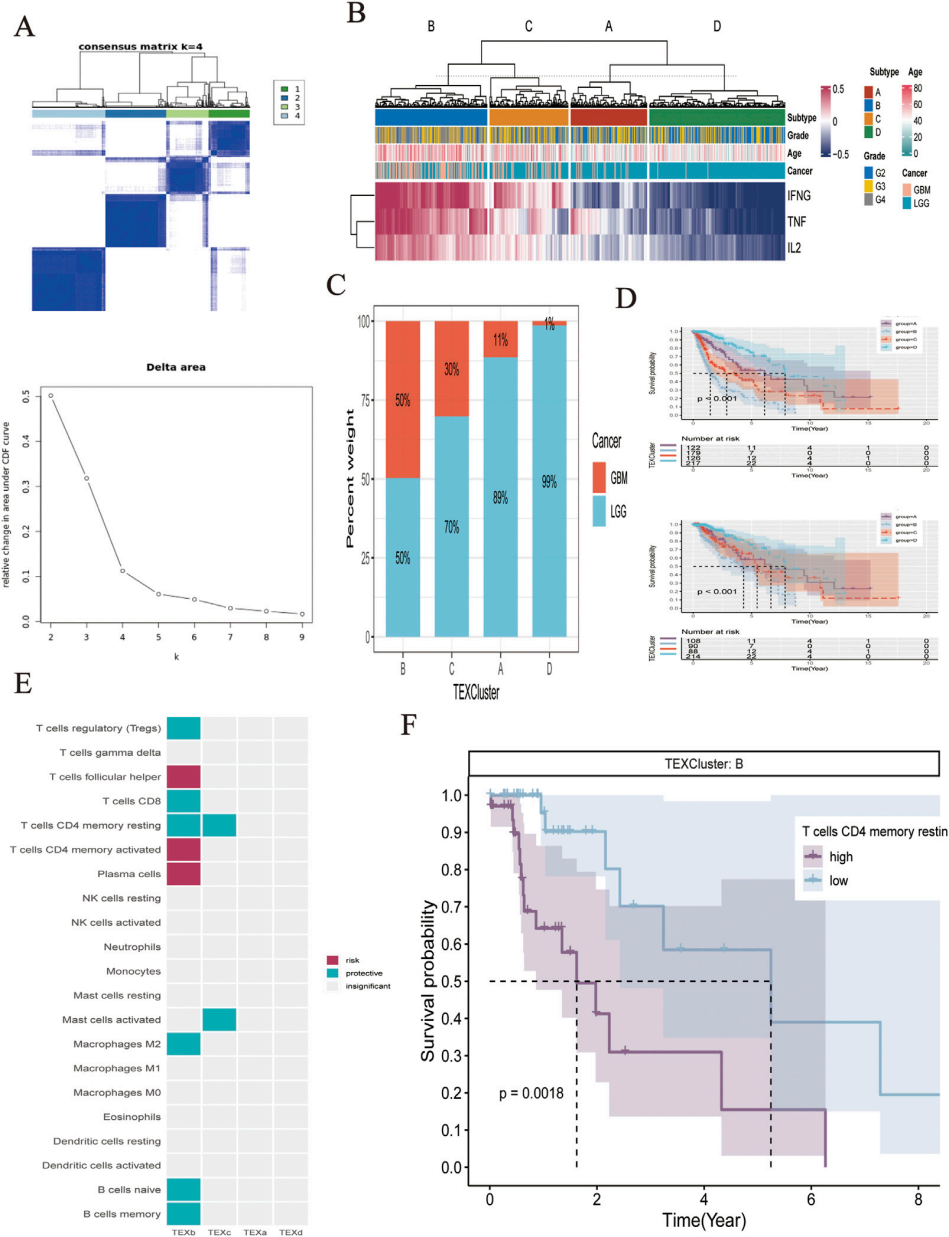
**(A)** Unsupervised clustering classification. **(B)** Heatmap showed the expression of IL2/IFNG/TNFA pathway in different subtypes and clinical pathology. **(C)** Bar chart showed the proportion of LGG and GBM in different molecular subtypes. **(D)** K-M analysis of four subtypes. **(E)** The effect of different immune cell subsets in tumor microenvironment on the clinical outcome of glioma. **(F)** Effect of infiltration level of T cells CD4 memory resting on clinical outcome.

First, the three pathways of IL2, IFN-γ, and TNFα were assessed for their Gene Set Variation Analysis (GSVA) scores according to the HALLMARK gene set, based on which unsupervised clustering analysis was conducted. The delta results showed that grouping into four clusters yielded the best results (Figure 1A). The heat map illustrates the expression levels of IL2, IFN-γ, and TNFα pathways in different subtypes, and we found that the pathways of IL2, IFN-γ, and TNFα all presented a state of high expression in cluster B (Figure 1B). Subsequently, the proportion of glioma and LGG patients in different TEXcluters was examined, with GMB patients accounting for the highest proportion (50%) in Cluster B and only 1% in Cluster D (Figure 1C). Meanwhile, the predictive efficacy of their TEX groupings was tested, all of which showed that patients in Cluster D had the best survival status, whereas patients in Cluster B had the worst survival (Figure 1D). The immune cell infiltration component of the tumor microenvironment of the four cluster patients was assessed using the CIBERSORTX algorithm, and Cox regression analysis was performed to investigate the prognostic value of a wide range of cells in each TEXCluster patient. Among them, resting T cell memory was found to be a protective factor in both TEXb and TEXc (Figure 1E). Therefore, resting T cell memory was grouped according to its level of infiltration and tested to predict clinical outcomes in glioma patients. Intriguingly, patients with high T cell memory resting infiltration had poorer survival prognosis (Figure 1F). Therefore, further analysis was conducted.

### Identification of T cell exhausted related genes

**FIGURE 2:**
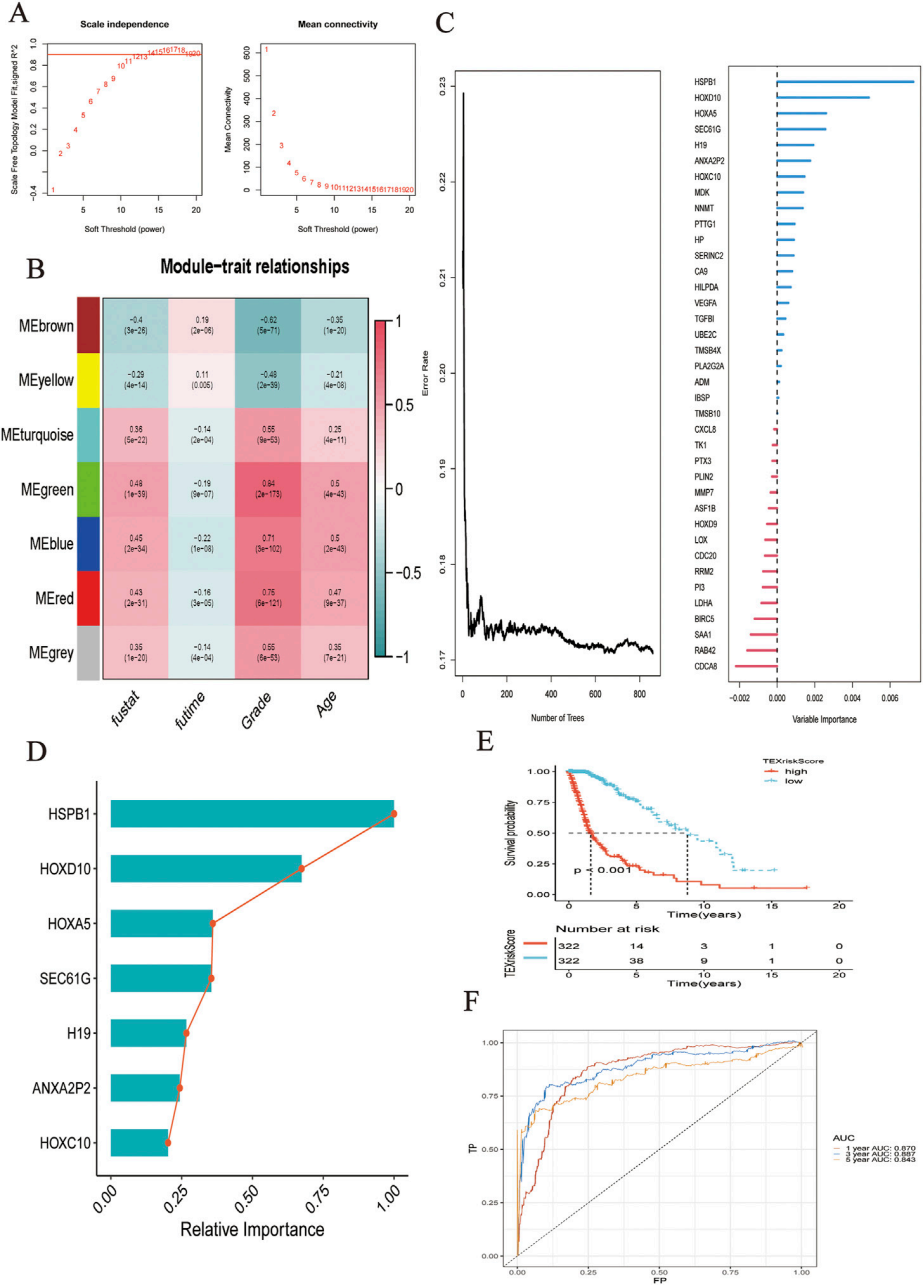
**(A)** Network topology analysis of various soft threshold power. **(B)** Module-clinical feature association: Each row corresponded to a module feature gene, and each column corresponded to a clinical feature. **(C)** Survival random forest analysis was conducted to assess the importance of variables. **(D)** Seven genes were most related to survival. **(E)** K-M analysis showed the predictive performance of the model. **(F)** The ROC curve showed the accuracy of the prediction model in predicting 1-, 3-, and 5-year survival status.

Differential expression analysis was performed on patients with TEXCluster 4, and concatenation was performed for WGCNA analysis. The WGCNA method was used to screen for clinically relevant genes, and a weighted gene co-expression network was constructed using the following parameters: power of *β* = 4 and scale-free R2 = 0.9. Consequently, seven color modules were finally obtained by merging similar modules, and the MEgreen group was found to be the most relevant to survival-related information, after which MEgreen was selected for subsequent analysis (Figures 2A,B).

### Construction of TEXScore prediction model

Based on the selected gene sets and corresponding clinical outcomes, we performed survival random forest analysis, an algorithm was used to process right-censored survival data. When Ntree = 859, the error rate was the lowest, and the seven genes most associated with clinical outcomes were *HSPB1, HOXD10, HOXA5, SEC61G, H19, ANXA2P2*, and *HOXC10* (Figure 2C). The importance of these genes in predicting the clinical outcome of patients with glioma is shown in Figure 2D. The LASSO regression algorithm was used to construct the model.

$$risk\;score={\;}^{\prime \prime }0.4956\times HSPB1+0.1231\times HOXD10+0.0697\times HOXA5+0.0037\times \mathrm{SEC}61G+0.0911\times H19+0.145\times ANXA2P2+0.064\times HOXC10$$

**FIGURE 3:**
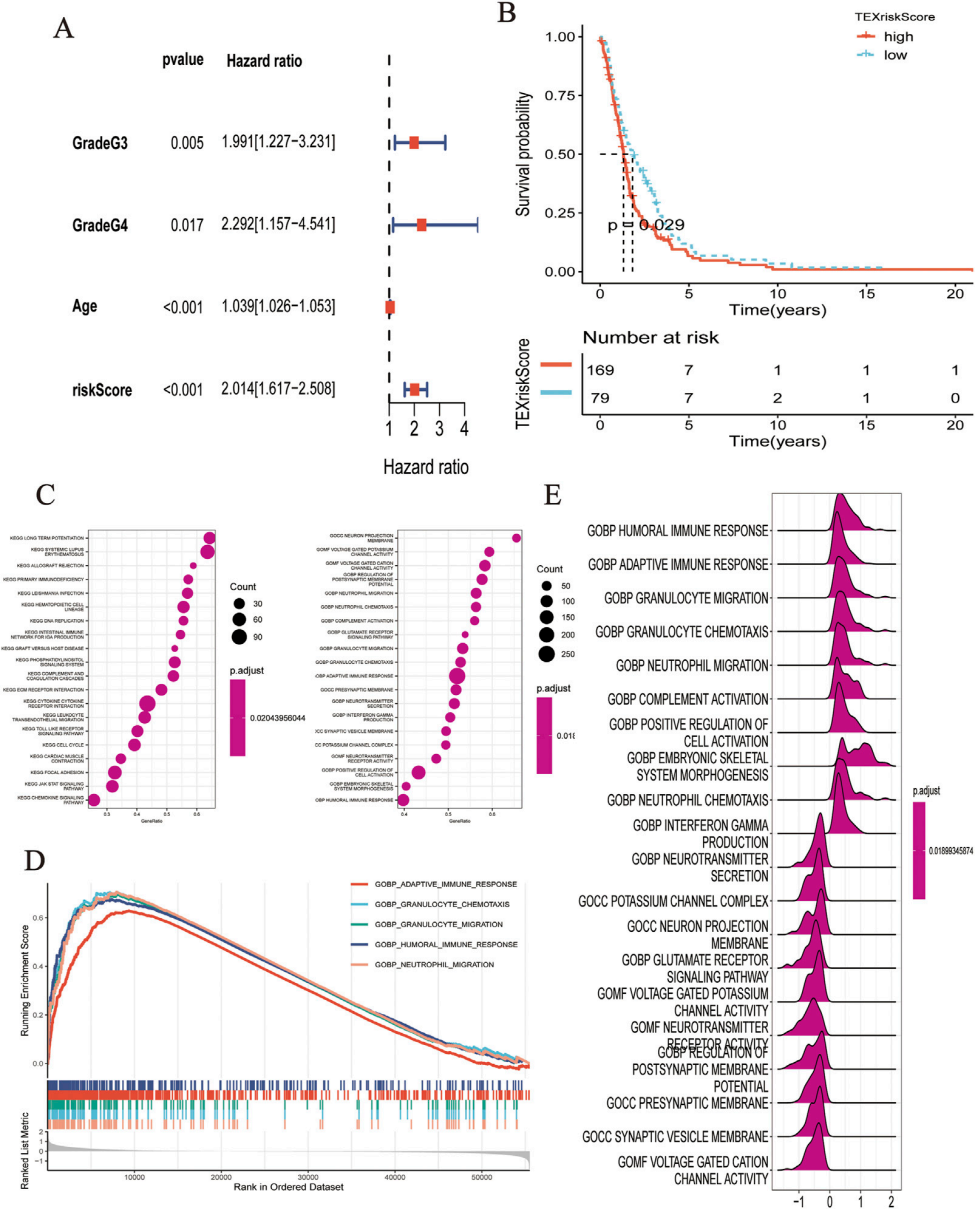
**(A)** Multivariate COX regression analysis demonstrated that RiskScore was an independent predictor. **(B)** K-M analysis showed the predictive performance of the model in external cohort. **(C–E)** Biological function analysis based on GO dataset.

As shown by the Kaplan–Meier curve, patients with a low TEXScore had a significantly higher OS rate than those with a high TEXScore (*p* < 0.001), suggesting that the overall survival time of patients would decrease with increasing risk score (Figure 2E), and the ROC curve indicated that its performance in predicting survival rate at 1, 3, and 5 years was 0.870, 0.887, and 0.843, respectively (Figure 2F). It was found by the multivariate COX regression analysis that TEXSore was an independent factor for prognosis with a Hazard ratio of 2.014 (Figure 3A). An external dataset was used to validate the predictive performance of the TEXScore model, and the Kaplan–Meier survival results were in agreement with the training set (Figure 3B). We then detected the expression of HSPB1, the most important gene, in the normal human astrocyte cell line (HEB) and GBM cell line (U251), and found significant differences. HSPB1 was expressed at higher levels in U251 cells than in HEB cells (Supplementary Figure S1). These results indicated that HSPB1 plays an important role in glioma progression.

### Exploration of the correlation between TEXScore and biological function

**FIGURE 4:**
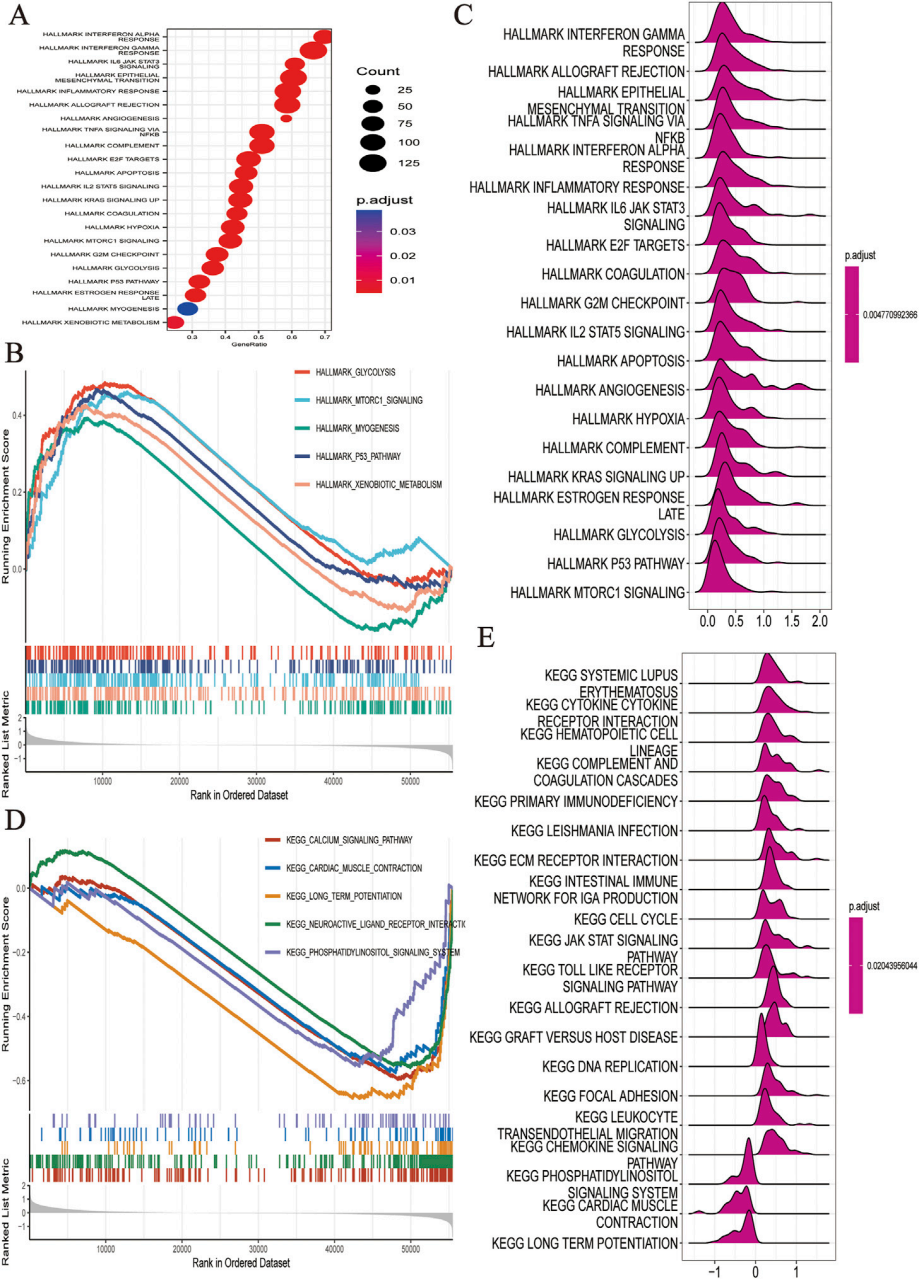
**(A–C)** Biological function analysis based on HALLMARK dataset. **(D,E)** Biological function analysis based on KEGG dataset.

Based on the HALMARK, KEGG, and GO datasets, we explored the correlation between TEXScore and biological function in patients with glioma. First, we found that the TEXScore was correlated with tumor pathways, including positive regulation of cell activation, focal adhesion, and the JAK-STAT signaling pathway. The TEXScore was also highly correlated with toll-like receptor signaling, granulocyte migration pathway, adaptive immune response, and other immune pathways (Figures 3C–E). Both the HALMARK and KEGG gene sets confirmed these results (Figure 4).

**FIGURE 5:**
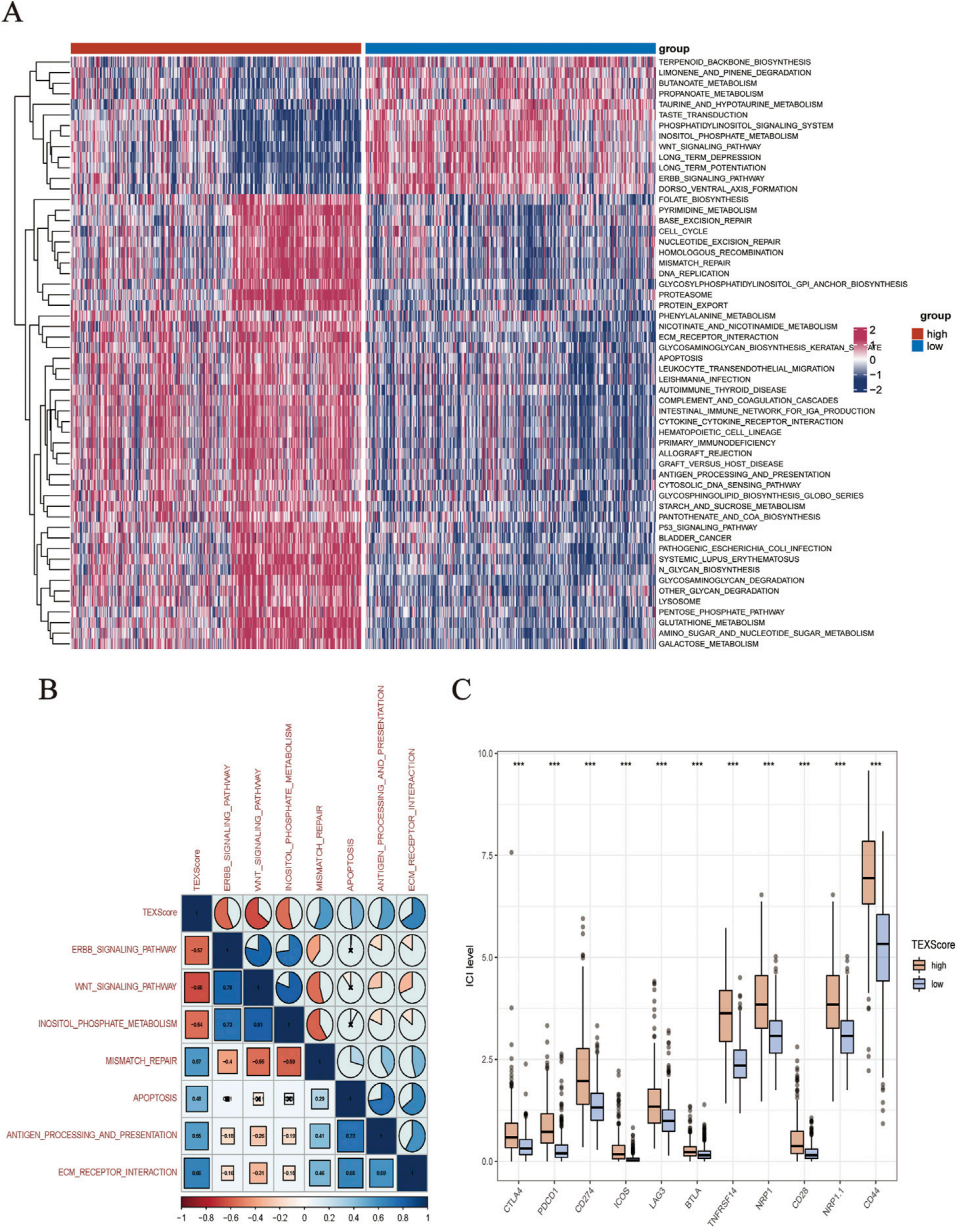
**(A)** Heatmap showed the enrichment of GSVA analysis (The enrichment degree of each patient in tumor formation and other related pathways). **(B)** Correlation between TEXScore and pathways. **(C)** Immunomodulator expression levels in different TEXScore groups.

The levels of pathway enrichment in patients with high and low TEXScore are illustrated in the heatmap (Figure 5A). Based on the above GSEA results, several gene sets were selected for GSVA analysis, and a negative correlation was found between TEXScore and tumor formation and other pathways, such as the ERBB signaling pathway and WNT signaling pathway, but a positive correlation with mismatch repair, antigen processing, and presentation (Figure 5B).

### Correlation of TEXScore with immune checkpoints

**FIGURE 6:**
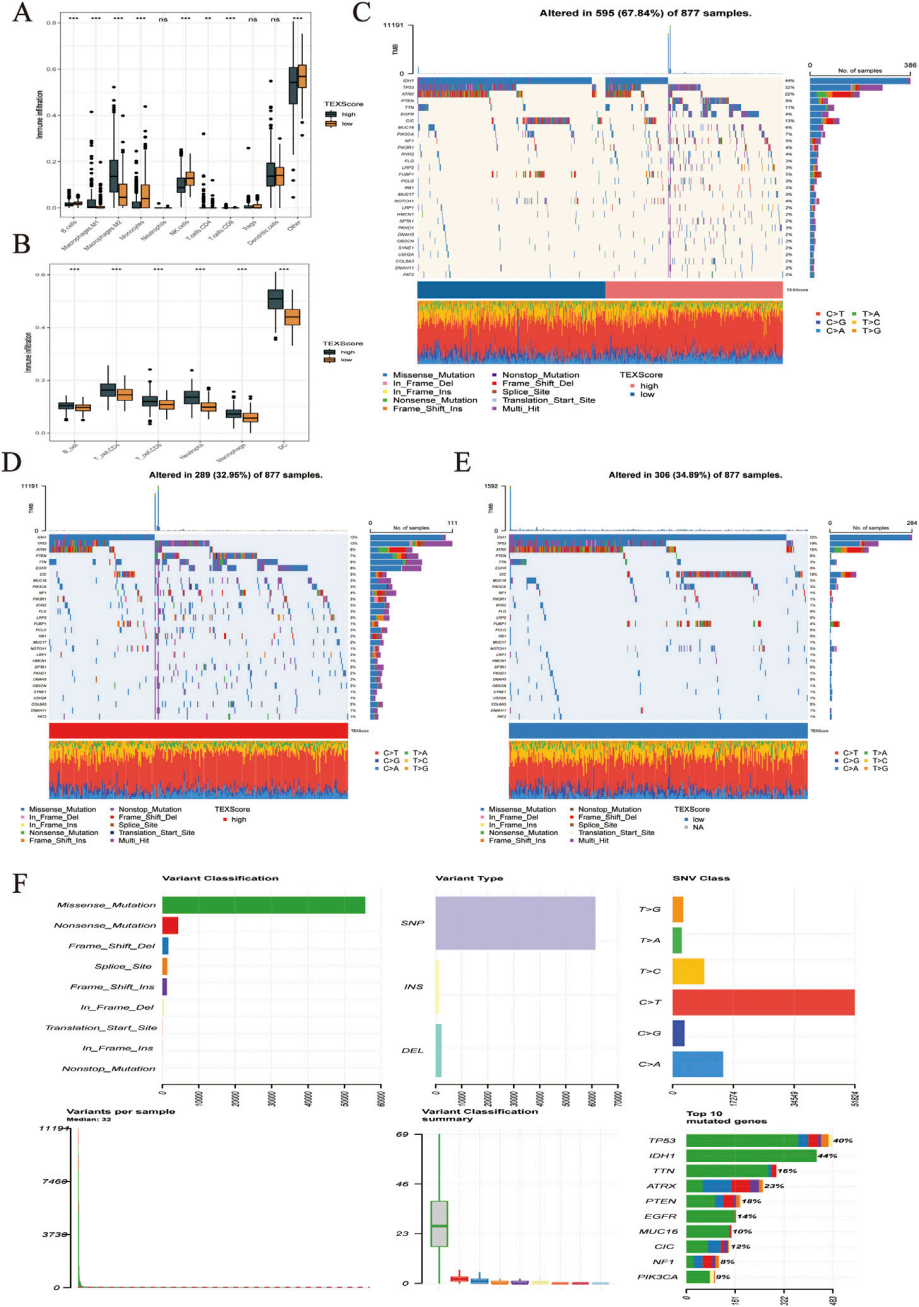
**(A,B)** Infiltration levels of immune cell subsets in different TEXScore groups. **(C–F)** High and low TEXScore patients with its mutation landscape.

To check the effectiveness of the TEXScore in predicting the clinical response to immunotherapy in glioma patients, the expression of immune checkpoints in patients with high and low TEXScore was examined. The results showed that immune checkpoint expression was higher in patients with a high TEXScore than in those with a low TEXScore (Figure 5C). Subsequently, the degree of immune cell infiltration in patients in the high and low TEXScore groups was calculated using quanTlseq and TIMER algorithms. The quanTlseq algorithm revealed that the M2 macrophage component was significantly enriched in the high TEXScore patients, whereas immune effector cells were predominant in the low-risk group (Figure 6A).

The TIMER algorithm showed a higher proportion of both immune effector cells and M macrophages in the high-risk group, which sheds light on the subsequent analysis of whether the immune effector cells in the high-risk group were in a state of immune depletion (Figure 6B). The mutations in LGG and glioma are shown in Figure 6C. Figures 6D,E present the mutation landscape of the high and low TEXScore patients, respectively, with the high TEXScore group having a lower mutation rate than the low TEXScore group. The overall mutation landscape is illustrated in Figure 6F, including variant classification, mainly composed of missensemutation, variant type, mainly composed of SNPs, SNV class, mainly composed of C > T, and variants per sample, Mediant:32.

**FIGURE 7:**
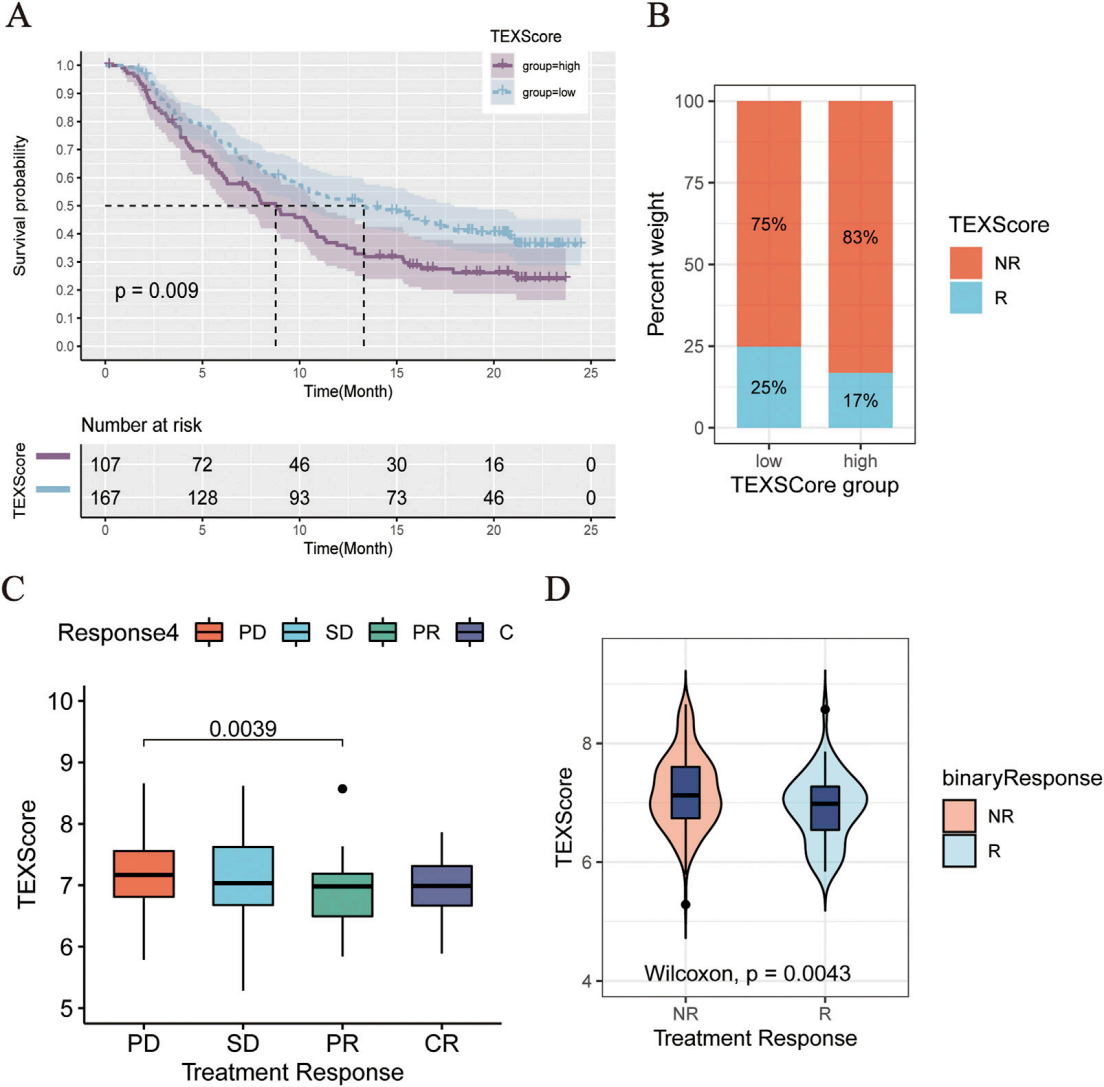
**(A)** Comparison of clinical outcomes between high and low TEXScore patients. **(B)** Bar chart showed the effectiveness of immunotherapy in patients with high and low TEXScore. **(C,D)** TEXScore corresponding to the clinical response of patients to immunotherapy.

Data from IMvigor210 were then obtained to validate the performance of the TEXScore model in predicting the effect of immunotherapy. First, the patients were categorized into high- and low-risk groups according to the TEXScore formula. The Kaplan–Meier survival model proved the effectiveness of TEXScore in predicting the clinical outcome of patients, with patients with a high TEXScore experiencing worse survival status than those with a low TEXScore (Figure 7A). Following this, the bar chart demonstrates that patients with low TEXScore responded more significantly to immunotherapy (Figure 7B), suggesting that the lower the TEXScore, the better the patients responded to immunotherapy, with PR and CR patients having the lowest TEXScore (Figures 7C,D).

## Discussion

A growing body of research suggests that rather than being an immune-privileged organ, there are many innate and adaptive immune responses within the central nervous system (von Roemeling et al., 2020; Broekman et al., 2018; Schmitt et al., 2021). A complex tumor immune microenvironment in gliomas is formed by tumor cells, stromal cells, immune cells, and extracellular matrix (Hambardzumyan et al., 2016; De Boeck et al., 2020). T cells, a class of immune cells that are essential for the immune response in gliomas, play a key role in tumorigenesis and progression (Quail and Joyce, 2017; O'Rourke et al., 2017). As a result, T cells appear to be key to glioma immunotherapy. Reportedly, significant heterogeneity in T cell depletion exists between different grades of gliomas and between samples of the same grade (Woroniecka et al., 2018; Sadik et al., 2020; Kreatsoulas et al., 2022). To this end, seven T cell depletion-related genes with prognostic value in gliomas were filtered out, based on which a TEXScore model was constructed. As the results suggest, significant differences exist in the risk assessment of gliomas of different grades, subtypes, and molecular characteristics. In particular, a more complex tumor immune microenvironment in glioma patients usually predicts a far more interior prognosis. According to this study, higher risk assessment scores were associated with stronger immune responses, a more complex tumor immune microenvironment, and a worse prognosis for glioma patients. It has been indicated in several studies that while TEXScore positively correlates with certain immunotherapeutic targets and is enriched in hot tumors (positively correlated with immune cell infiltration), it also correlates with T cells depletion markers, such as *HSPB1* and *HOXA5*, suggesting that the stronger the suppression of antitumor immunity, the greater the depletion of T cells in gliomas. Recent studies have also revealed the failure of depleted T cells to recover their tumor-killing capacity. The findings of this study were further validated in the IMvigor 210 immunotherapy dataset, as patients with a low TEXScore proved to significantly benefit from immunotherapy. Consequently, the development of a TEXScore model would contribute to the effective prediction of prognosis and immunotherapy effects in patients with glioma.

Random forest analysis and LASSO regression analysis were employed to construct the risk assessment related to T cell depletion in glioma, a pioneering practice. The generated TEXScore may provide a promising assessment of the prognosis and tumor immune microenvironment among glioma patients, and because of its well-validated performance in other cohorts, it may serve well for clinical translation. Heat shock protein beta-1 (HSPB1) is a negative regulator of iron cancer cell death, and *HSPB1*, as a new regulator of iron cancer cell death in previous experiments, plays an important role in iron-mediated cancer therapy (Sun et al., 2015). *HOXD10*, *HOXA5*, and *SEC61G* have all been shown to play an important role in breast tumorigenesis and were identified as potential biomarkers for the diagnosis of breast cancer (Stasinopoulos et al., 2005; Vardhini et al., 2014; Ma et al., 2021). In addition, *SEC61G* was identified as a novel prognostic marker to predict survival and treatment response in glioblastoma patients in recent studies (Liu et al., 2019). H19 has been shown to play an important role in the tumorigenicity and stemness of glioblastoma and may be a therapeutic target for the treatment of glioblastoma in the future (Jiang et al., 2016). In a cell experiment, pseudogene ANXA2P2 knockdown showed its tumor suppressor function by inhibiting the PI3K/PKB pathway in glioblastoma cells (Ni et al., 2021). *HOXC10* belongs to the homeobox gene family, which encodes a highly conserved family of transcription factors that play an important role in morphogenesis in all multicellular organisms. Similarly, *HOXC10* overexpression promotes angiogenesis in human gliomas through interaction with PRMT5 and upregulation of VEGFA expression *in vitro* (Tan et al., 2018; Guan et al., 2019). These results suggest that our selected prognostic genes play an important role in glioma or cancer and are sufficient to demonstrate the stability of prognostic models. Currently, the efficacy of immunotherapy for glioma remains unclear, and there are no clinical trials on the potential benefits of immunotherapy in glioma patients. With our TEXScore, the T cell depletion levels of glioma patients can be effectively assessed with differentiated immunotherapy-predicted outcomes, as validated by the validation cohort and IMvigor 210 immunotherapy dataset. Furthermore, the TEXScore provides certain predictive values for immunotherapy treatment of glioma patients, which may serve as a fundamental basis for the treatment of glioma patients for further clinical applications in the future.

In the present study, TEXScore was found to be correlated with positive regulation of cell activation, focal adhesion, JAK STAT signaling pathway, and other tumor pathways, and significantly correlated with Toll-like receptor signaling, granulocyte migration pathway, adaptive immune response, and other immune pathways. In addition, there was a negative correlation between TEXScore and pathways, such as tumor formation, including ERBB signaling pathway and WNT signaling pathway, and a positive correlation with mismatch repair, antigen processing, and presentation, indicating the effectiveness of risk assessment scores in describing the relative status of tumor activation pathways and antitumor immune depletion in glioma samples. The risk assessment score was effective in describing the relative status of tumor activation pathways and antitumor immune depletion in glioma samples. Although several previous studies have been conducted on the prediction of immunotherapy response among glioma patients, hundreds of genes were used as test subjects. However, with our TEXScore, the response of patients in the IMvigor cohort to immunotherapy could be predicted by detecting only seven T cell depletion-related genes. We are convinced that the TEXScore may lead to promising clinical applications to facilitate the development of new glioma immunotherapies.

However, this study has several limitations. First, this was a retrospective study and no prospective study was performed for validation. Second, there is no public dataset of immunotherapy for glioma patients to validate immunotherapy outcomes. In addition, the interaction between T cell depletion and tumor cells should be further investigated in combination with single cell sequencing. Finally, while we validated the relative expression of HSPB1 using qRT-PCR analysis, it is necessary to thoroughly investigate the mechanisms by which these seven genes modulate T cell depletion in gliomas in order to better integrate risk assessment and clinical practice. But our experiment remains somewhat superior, and we identify a reliable model composed of genes involved in T cell depletion, unlike other common prognostic models, by assessing global gene expression profiles, and the model is of great value in predicting glioma patient outcomes.

## Conclusion

In brief, upon the assessment of global gene expression profiles, a reliable TEXScore model consisting of seven genes related to T cell depletion was identified, which is of great value in predicting the prognosis of glioma patients and may help set targets for treating glioma patients.

## Data Availability

The original contributions presented in the study are included in the article/Supplementary Material, further inquiries can be directed to the corresponding author.
