# Supplementary figures and images for: T cell exhaustion assessment algorism in tumor microenvironment predicted clinical outcomes and immunotherapy effects in glioma
Source: Front Genet. 2022 Dec 2;13:1087434. doi: 10.3389/fgene.2022.1087434 (PMC9755497; doi:10.3389/fgene.2022.1087434)

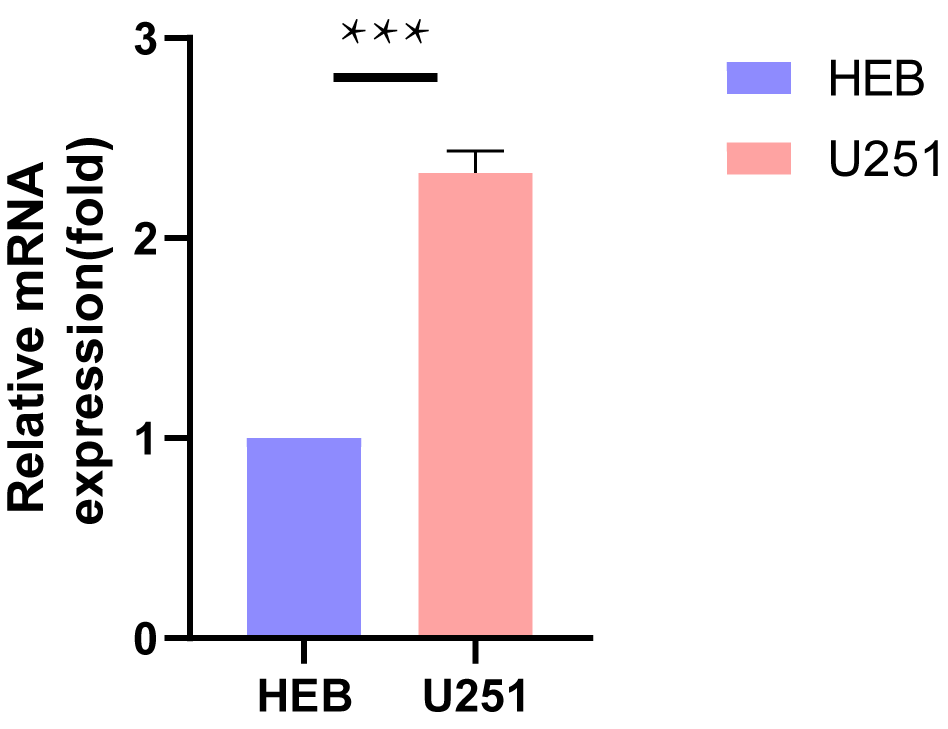


Figure S1| The expression level of HSPB1.

Supplement: Supplementary file 2 [file Table1.DOCX]
